# Impact of the COVID-19 pandemic on adult mental health-related admissions at a large university health system in North Carolina – one year into the pandemic

**DOI:** 10.1371/journal.pone.0293831

**Published:** 2023-12-21

**Authors:** Tatyana Der, Nicole Helmke, Jason E. Stout, Nicholas A. Turner

**Affiliations:** 1 Department of Medicine, Duke University School of Medicine, Durham, North Carolina, United States of America; 2 Department of Psychiatry and Behavioral Sciences and Department of Medicine, Duke University School of Medicine, Durham, North Carolina, United States of America; 3 Division of Infectious Diseases, Duke University School of Medicine, Durham, North Carolina, United States of America; University of Manitoba Max Rady College of Medicine, CANADA

## Abstract

**Objective:**

Pandemic-associated stress may have exacerbated preexisting mental health and substance use disorders (MH/SUD) and caused new MH/SUD diagnoses which would be expected to lead to an increase in visits to emergency departments and hospital admissions for these conditions. This study assessed whether the proportion of hospital and emergency department encounters for MH/SUD diagnoses increased during the first year of the COVID-19 pandemic in the United States.

**Methods:**

We conducted a longitudinal (interrupted time series) analysis of 994,724 eligible encounters identified by electronic query between January 1, 2016 and March 31, 2021. Of these, 55,574 encounters involved MH/SUD diagnosis. The pre-pandemic period was defined as January 1, 2016 to March 31, 2020, and the pandemic period was defined as April 1, 2020 to March 31, 2021. All statistical analyses were performed with R.

**Results:**

No significant trend in MH/SUD encounters at baseline (rate ratio 1.00, 95% CI 0.99–1.01, p = 0.75) was observed. However, the onset of the pandemic was temporally associated with a significant level increase in the proportion of MH/SUD encounters relative to overall encounters (rate ratio 1.14, 95% CI 1.06–1.21, p<0.001) with no change in the overall trend (rate ratio 0.99, 95% CI 0.90–1.10, p = 0.89).

**Conclusions:**

The significant pandemic-associated increase in the proportion of MH/SUD encounters relative to overall encounters was driven largely by sustained numbers of MH/ SUD encounters despite a decrease in total encounters. Increased support for mental health care is needed for these vulnerable patients during pandemics.

## Introduction

The impact of the COVID-19 pandemic on populations made vulnerable by systemic inequity, including people suffering from mental health (MH) and substance use disorders (SUD), is expected to be significant due to disruption of health care access and social isolation stemming from various public health measures designed to decrease the transmission of disease. Pre-existing MH disorders have been associated with higher mortality risks, more severe illness course and lower access to intensive care with COVID-19 infection [1,2]. For people with SUDs, social isolation can lead to the collapse of support structures, increasing risk of relapse as well. This was observed nationally, with increases in drug overdose, alcohol sales and consumption during the pandemic [3]. An increase in alcohol use during the pandemic may have led to an increase in cases of new alcohol-related liver injury in addition to an increase in alcohol-related psychiatric disorders. Pandemic-associated stress and a generalized increase in anxiety and depression [4–6] may have exacerbated preexisting MH disorders and caused new MH/SUD diagnoses which would be expected to lead to an increase in visits to emergency departments (EDs) and hospital admissions for these conditions.

Data on ED and hospital admissions for psychiatric disorders during the pandemic remain inconsistent with the majority of studies conducted internationally. For example, the studies from Europe, UK, Australia, South Korea and Canada variably reported both increases or decreases in visits [7–18]. In the United States, there were inconsistent and limited data on ED visits and admissions for adults in Midwest, rural areas of West Texas, urban Kentucky and New York City as well [19–22]. The variability in psychiatric services patterns across studies could be attributed, in part, to different characteristics of lockdowns in different countries and states, changes in access to health care during lockdowns, limited and varied number of diagnoses studied. Few studies extended more than 6 months beyond the start of the pandemic, limiting their ability to fully assess seasonality and longer-term consequences of pandemic-related stressors.

We sought to evaluate the temporal relationship between the COVID-19 pandemic and adult hospital encounters (ED visits and hospitalizations, combined) for MH/SUD diagnoses in our health system to better understand the impact of the COVID-19 pandemic on mental health and the health system as a whole. We hypothesized that after adjusting for secular trends, the rate of hospital and ED encounters for MH and SUD diagnoses would be greater during the first year of the pandemic (April 1, 2020 to March 31, 2021) than during prior years. We also hypothesized that the proportion of patient encounters with preexisting MH/SUD encounters would be greater during the pandemic period compared to pre-pandemic.

## Methods

We conducted a longitudinal (interrupted time series) analysis to examine the effect of pandemic-related factors on MH and SUD related hospital encounters, including admissions and ED visits, for all adult patients (18 years and older) evaluated within the Duke University Health System (DUHS) hospitals (Duke University Hospital, Duke Regional Hospital and Duke Raleigh Hospital) between January 1, 2016 and March 31, 2021. Duke University hospital is a tertiary referral center with 1,048 inpatient beds and its own ED equipped to treat 90,000 patients per year. Duke Regional Hospital serves surrounding counties and has 388 inpatient beds, its own ED, now able to accommodate over 60,000 patients per year. Duke Raleigh Hospital is a community hospital with 186 inpatient beds and its own ED treating over 40,000 patients per year.

For the purpose of this analysis, the pre-pandemic period was defined as January 1, 2016 to March 31, 2020, and the pandemic period was defined as April 1, 2020 to March 31, 2021. These analysis periods were chosen based on the timing of the statewide “Stay at Home Order” in North Carolina, implemented on March 27, 2020. The durations of pre-pandemic and pandemic periods were chosen to assess the possible effect of seasonality and to give more data on secular trends that existed before COVID (for example, to account for state legislative interventions to address mental health and substance use in North Carolina) [23–26].

A mental health or substance use-related encounter was defined as an ED visit or hospital admission in which either the primary reason for ED visit or the final primary diagnosis, designated by International Classification of Diseases, Ninth or Tenth Revision (ICD-9/10) codes, was included in the **S1 Table.** DEDUCE™ V8 query tool [27] was used to identify relevant DUHS encounters and all-cause (Total) encounter counts. DEDUCE is a well-validated interface for querying Duke University Health System’s electronic medical record data. Diagnostic and clinical information is recovered as entered by clinicians. Personal and demographic data are recovered as self-reported by patients at time of presentation. For the time periods of interest, we queried all inpatient adult (age ≥18 years) admission and ED visits within Duke Health System for accompanying ICD9/10 diagnosis codes. For MH/SUD related encounters, we additionally queried demographic data (age, race, sex). As we were interested in the impact of the pandemic on mental health and the health system overall, we used encounters as the unit of interest–including both ED visits and hospital admissions. To avoid duplication, ED visits resulting in admission were treated as a single encounter. If the same patient presented more than once within the study period, each encounter was counted separately.

Patients were determined to have a preexisting MH or SUD history if they had a previous encounter anywhere within the Duke Health System with one of these diagnoses from **S1 Table** between July 1, 2013 (the first available year in DEDUCE™ V8 query tool) and March 31, 2021. Once a patient had their first encounter for MH/SUD diagnosis, any subsequent encounter would classify the patient as having preexisting MH/SUD history. While manual chart review was used for a small proportion of encounters to assure query accuracy, MH/SUD history was not individually verified due in part to the shear size of the dataset. Additionally, both electronic and manual queries carry the same misclassification risk for subjects receiving a first diagnosis outside of our health system. All adult patients with selected diagnoses of interest were included. Encounters lacking associated demographics were excluded (n = 25). This study was approved by the DUHS institutional review board.

### Statistical analysis

Interrupted time series regression was used for the analysis of the primary outcome. Trends in the number of encounters by calendar year were displayed graphically. The numbers of all-cause (Total) and MH related encounters were summarized by month and calendar year, and descriptive statistics of the patient cohort (age, sex, and race) were obtained. The number and proportion of patient encounters with pre-existing MH and SUD encounters were summarized by time period (pre-pandemic or pandemic). The model included time, pandemic, and time after pandemic terms to permit assessment for both level and slope changes temporally associated with COVID-19 [28]. Total encounters was used as an offset term adjusting for overall volume of healthcare encounters. Rate ratios for baseline and overall trends were normalized to change per 12-month period to make effect estimates more readable. There was evidence of overdispersion in the data so we chose the negative binomial model. Model fit was ascertained using standard generalized linear regression diagnostics. Inspection of QQ plots suggested a negative binomial distribution to be reasonable. We found no significant evidence for autocorrelation using the Breusch-Godfrey test [29]. We assessed for potential seasonality [28] as well, both visually and with addition of seasonal terms, but found no evidence for any significant effect. Fitted vs actual plots were constructed as a final visual inspection of each model’s accuracy. No formal power calculations were conducted due to lack of standardized methods for power analysis of interrupted time series regression [28,30]. For the secondary hypothesis we used the homogeneity of proportions test for conditional probabilities. An α value of 0.05 was set as the threshold for statistical significance, and all tests were 2-tailed. All statistical analyses were performed with R Studio version 2021.09.1 and R version 4.0.2 (R Project for Statistical Computing).

## Results

There were a total of 994,724 all-cause inpatient and emergency department encounters between January 1, 2016 and March 31, 2021–169,815 of them occurred during the pandemic period and 824,909 during the pre-pandemic period. After removing 25 of the encounters with missing age data, 55,574 (5.6%) encounters among 25,579 unique patients involved MH and SUD diagnoses. During the pandemic period alone, there were 10,433 encounters for MH/SUD diagnoses, which constituted 6.1% of the total encounters during that time **(Table 1).**

**Table 1:** Demographic and clinical data on patient encounters with mental health encounters in Duke University Health System between January 1, 2016 and March 31, 2021.

	Pre-pandemic period (Total encounters: 824,909)		Pandemic period (Total encounters: 169,815)		Total Cohort of Mental Health encounters (Total encounters 994,724)	
	N	%	N	%	N	%
**Mental health and substance abuse encounters**	45141	5.5	10433	6.1	55574	5.6
**Age (mean(SD))**	45(16.8)		44(16.5)		45(16.7)	
**Female sex**	21573	47.8	4557	43.7	26130	47.0
**Race—Asian/Pacific Islander**	743	1.6	147	1.4	890	1.6
**Race—Black**	18012	39.9	4502	43.2	22514	40.5
**Race—Caucasian**	23330	51.7	4959	47.5	28289	50.9
**Race—Other**	3056	6.8	825	7.9	3881	7.0
**With prior MH/SUD encounter (N, % of patient encounters)**	22139	49.0	5413	51.9	27552	49.6

**Footnotes**: Pandemic period: April 2020 to March 2021. Pre-pandemic period: January 2016 to March 2020.

**Fig 1:**
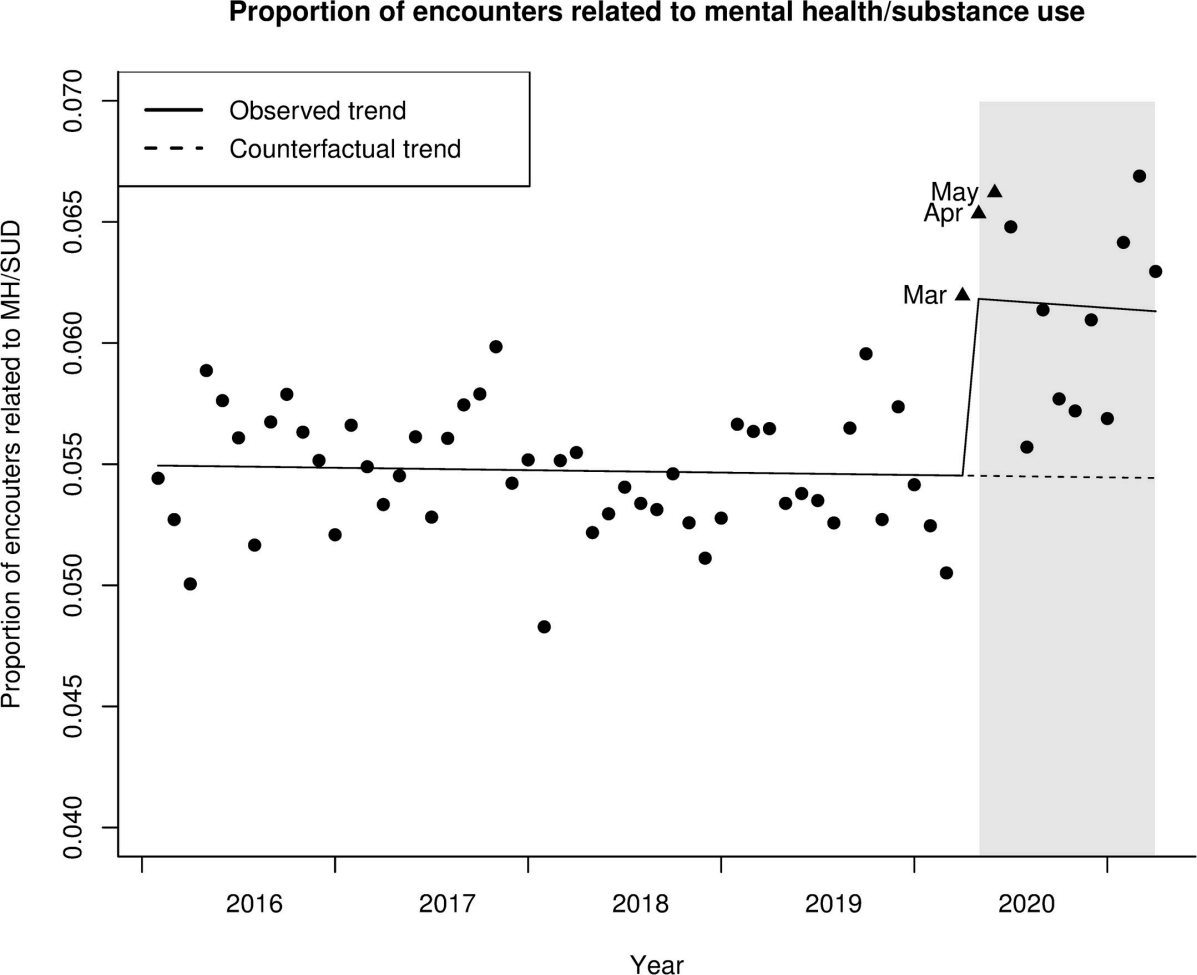
Plot of the proportion of MH/SUD encounters relative to total encounters, 1/2016-3/2021.

The raw numbers of total all-cause and mental health encounters during the study period are shown in **S1 and S2 Figs**. While both all-cause and MH/SUD encounters decreased at the onset of the pandemic, the proportion of MH/SUD encounters increased in March 2020, peaking at 6.6% in May 2020, remaining above 6% for the next month, and only dropping down to 5.6% by July 2020, as depicted in **Fig 1**.

This study found no significant trend in MH/SUD encounters during the baseline period (rate ratio 1.00, 95% CI 0.99–1.01, p = 0.75). However, the onset of the pandemic was temporally associated with a significant level increase in the proportion of encounters related to MH/SUD relative to total encounters (rate ratio 1.14, 95% CI 1.06–1.21, p<0.001). Furthermore, there was no significant change in overall trend (rate ratio 0.99, 95% CI 0.90–1.10, p = 0.89), **Fig 1**.

A post-hoc exploratory sensitivity analysis was performed for the primary outcome, to ensure that the potential outlier months of March-May 2020 were not overly influential. Coefficients and significance level for pre- and post-intervention trends remained robust, and the coefficient for level change attenuated to 8% but remained significant (1.08, 95% CI 1.03–1.13).

Of the 55,574 encounters involving MH/SUD diagnoses, 27,552 (49.6%) encounters involved patients with prior encounters for MH/SUD. The proportion of patient encounters with preexisting mental health encounters was significantly higher during the pandemic as compared to pre-pandemic (0.52 vs. 0.49, difference = 0.03; 95% CI 0.02–0.04; p<0.001).

## Discussion

While raw numbers of total and MH/SUD related encounters decreased beginning in April 2020, we observed a modest increase in the rate of MH/SUD related encounters relative to total encounters temporally associated with the onset of the pandemic. This pattern suggests that the increase in the proportion of MH/SUD related encounters was driven by a greater decrease in overall encounters than in MH/SUD related encounters—rather than a specific increase in the absolute number of MH/SUD encounters. The observed increase in the proportion of encounters related to MH/SUD needs during the COVID-19 pandemic is unfortunately not surprising based on the reported increased prevalence of anxiety and depression, substance use and suicidal ideations in the community [5,31], and could have been driven by pandemic-related stress and disrupted access to medical care during the pandemic. Prior research has suggested increased prevalence of anxiety, depression and substance use in the community during early pandemic [4–6], but also hesitancy to seek medical care due to fear of contracting COVID-19 or overloading the health system [32–35]. One possible explanation for a higher proportion of MH/SUD related encounters (despite a decrease in total encounters) might be that MH/SUD encounters really were either urgent or essential (rather than elective or optional). Many health care systems, including ours, paused elective surgeries and procedures early in the pandemic [36], which likely contributed to a decrease in total encounters–possibly contributing to the observed increase in the proportion of MH/SUD-related encounters. While difficult to measure, reduced access to elective and outpatient healthcare may have impacted psychological wellbeing as well, which is an area deserving of further study.

Our data builds on the evidence from Sacco [19] and Heppner [22]–similar to our results, both reported a drop in total ED encounters as the state of emergency was declared or coinciding with the peak of COVID-19 infections in the state. However, the proportion of encounters for select psychiatric diagnoses also increased. Shobassy, et al [20] also observed a decrease in total emergency psychiatry encounters during the pandemic period, but found a higher proportion required admission–perhaps suggesting greater severity at presentation. Additionally, their patient survey indicated the reduced access to mental healthcare during pandemic may have affected their decision to seek emergency care. This further supports a true increase in psychiatric need during the pandemic and highlights the importance of considering mental health needs as another consideration in the pandemic response.

Previous studies have shown that psychiatric patients wait longer in ED before receiving treatment compared to the general population [37,38], have longer lengths of stay which lead to crowding [39], decreased bed turnover, longer wait times and resultant decrease in financial revenue for health systems [38]. As mental health care has been historically underfunded [40] and fraught with structural inequities [41], the observed pandemic-associated increase in admissions and ED visits for psychiatric patients in this study is expected to further exacerbate disparities in care for these patients and strain hospitals. Strategies to explicitly address mental health needs, particularly for vulnerable populations, should be incorporated into future pandemic response plans.

As hypothesized, we observed a statistically significant though modest increase in the proportion of patients with previous MH/SUD history presenting for mental health crises during the pandemic. This was in contrast to other studies that noted increases in patients presenting for psychiatric emergency services with no prior history of psychiatric treatment [42,43]. This difference might be due to different definitions of preexisting psychiatric history, varying methods for ascertaining preexisting history, and different patient populations studied.

Our study has several important limitations. First, there is the potential for misclassification of the primary reason for the encounter due to coding errors but this is well-documented in the literature and does not prevent the use of electronic medical records [44]. Given that this is not a population-based study, it is possible that patients in the study area may have sought care at other medical centers outside of DUHS during the pandemic, leading to biases in the patient population. As our interest was focused on overall health system impacts, we did not separately assess trends in ED visits and admissions. Finally, the patient encounters were only enumerated and the underlying causes of each encounter were not evaluated in detail.

## Conclusions

The significant increase in the proportion of MH/SUD encounters at the onset of pandemic was driven largely by relatively sustained numbers of MH/ SUD encounters despite a decrease in total encounters. People with pre-existing mental health conditions were affected to a greater degree during the pandemic, though it is not clear if this was driven by pandemic-related stressors, changes in access to care, or other factors. Increased support for mental health care and further research are needed to identify the specific psychiatric conditions that constitute the majority of ED visits/admissions and the reasons why the proportion of mental health related admissions increased during the pandemic, in order to identify gaps in care and provide equitable access to care during future pandemics.

## Supporting information

S1 Fig Graph of total encounters over time, 1/2016-3/2021. (JPEG)

S2 Fig Graph of mental health encounters over time, 1/2016-3/2021. (JPEG)

S1 Table Grouping of diagnoses into following categories: Anxiety disorder, mood disorder (includes MDD and bipolar depression/mania), psychotic disorder, substance use, other. (DOCX)

S1 Dataset (XLSX)
