# Supplementary figures and images for: Impact of the COVID-19 pandemic on adult mental health-related admissions at a large university health system in North Carolina – one year into the pandemic
Source: PLoS One. 2023 Dec 21;18(12):e0293831. doi: 10.1371/journal.pone.0293831 (PMC10734981; doi:10.1371/journal.pone.0293831)

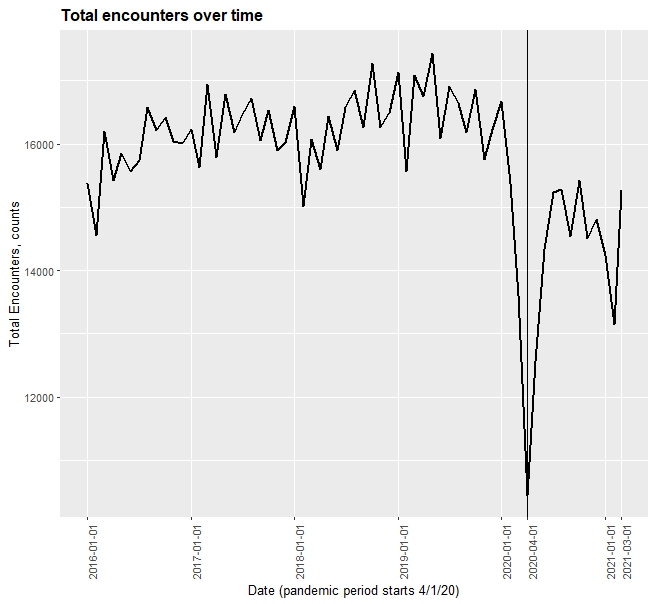

Supplement: S1 Fig — (JPEG) [file pone.0293831.s001.jpeg]

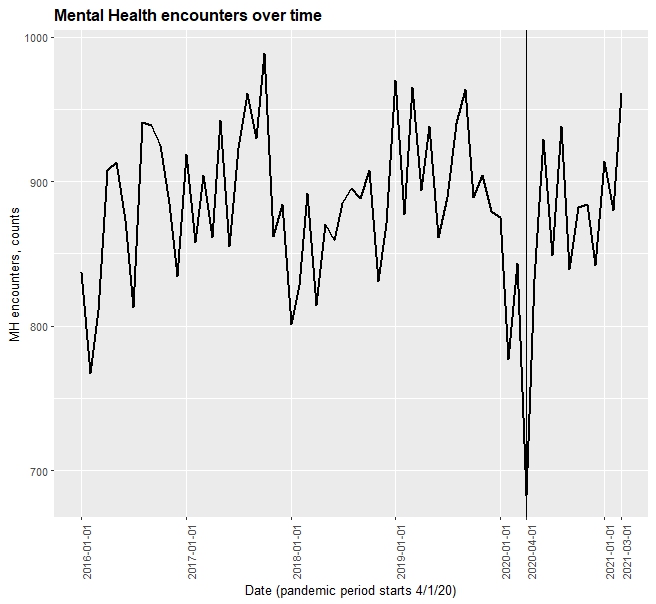

Supplement: S2 Fig — (JPEG) [file pone.0293831.s002.jpeg]
