# Supplementary material for: Impact of the COVID-19 pandemic on adult mental health-related admissions at a large university health system in North Carolina – one year into the pandemic
Source: PLoS One. 2023 Dec 21;18(12):e0293831. doi: 10.1371/journal.pone.0293831 (PMC10734981; doi:10.1371/journal.pone.0293831)
Supplement: S1 Table — (DOCX) [file pone.0293831.s003.docx]

**S1 Table**

Grouping of diagnoses into following categories: anxiety disorder, mood disorder (includes MDD and bipolar depression/mania), psychotic disorder, substance use, other.

**Anxiety disorder group, ICD-10/9 codes (63):**

| F40.0 | Agoraphobia, unspecified |
| --- | --- |
| F40.01 | Panic d/o with agoraphobia |
| F40.02 | Agoraphobia w/o panic d/o |
| F41.0 | Panic Disorder (episodic paroxysmal anxiety) |
| F41.1 | Generalized anxiety disorder |
| F41.3 | Other mixed anxiety d/o |
| F41.8 | Depression with anxiety |
| F41.9 | Anxiety disorder, unspecified |
| F41.9, F32.9 | Anxiety and depression |
| F42.8 | Neurosis, obsessive compulsive |
| F42.9 | OCD |
| F43.0 | Acute stress reaction |
| F43.10 | Post-traumatic stress disorder, unspecified |
| F43.11 | Post-traumatic stress disorder, acute |
| F43.20 | Adjustment disorder, unspecified |
| F43.21 | Adjustment disorder with depressed mood |
| F43.22 | Adjustment disorder with anxiety |
| F43.23 | Adjustment disorder with mixed anxiety and depressed mood |
| F43.29 | Adjustment d/o with physical complaints |
| F44.4 | Conversion d/o with abnormal movement |
| F44.5 | Pseudoseizure |
| F44.6 | Dissociative anesthesia |
| F44.7 | Mixed dissociative d/o |
| F44.81 | Multiple personality d/o |
| F44.9 | Conversion d/o |
| F45.1 | Undifferentiated somatoform d/o |
| F45.21 | Hypochondriasis |
| F45.22 | Body dysmorphic d/o |
| F45.41 | Somatoform pain d/o |
| F45.9 | Psychosomatic d/o |
| F48.1 | Depersonalization neurosis |
|  |  |
| 300.00 | Anxiety state, unspecified |
| 300.01 | Panic disorder without agoraphobia |
| 300.02 | Generalized anxiety disorder |
| 300.10 | Hysteria, unspecified |
| 300.11 | Conversion disorder |
| 300.12 | Dissociative amnesia |
| 300.13 | Dissociative fugue |
| 300.14 | Dissociative identity disorder |
| 300.15 | Dissociative disorder or reaction, unspecified |
| 300.16 | Factitious disorder with predominantly psychological signs and symptoms |
| 300.3 | Obsessive-compulsive disorders |
| 300.4 | Dysthymic disorder |
| 300.81 | Somatization disorder |
| 300.82 | Undifferentiated somatoform disorder |
| 300.89 | Other somatoform disorders |
| 300.9 | Unspecified nonpsychotic mental disorder (feeling suicidal, cutting own wounds, etc) |
|  |  |
| 308.0 | Predominant disturbance of emotions |
| 308.1 | Predominant disturbance of consciousness |
| 308.2 | Predominant psychomotor disturbance |
| 308.3 | Other acute reactions to stress |
| 308.4 | Mixed disorders as reaction to stress |
| 308.9 | Unspecified acute reaction to stress |
|  |  |
| 309.0 | Adjustment disorder with depressed mood |
| 309.1 | Prolonged depressive reaction |
| 309.24 | Adjustment disorder with anxiety |
| 309.28 | Adjustment disorder with mixed anxiety and depressed mood |
| 309.29 | Other adjustment reactions with predominant disturbance of other emotions |
| 309.3 | Adjustment disorder with disturbance of conduct |
| 309.4 | Adjustment disorder with mixed disturbance of emotions and conduct |
| 309.81 | Posttraumatic stress disorder |
| 309.82 | Adjustment reaction with physical symptoms |
| 309.9 | Unspecified adjustment reaction |

**Mood disorder (includes MDD and bipolar depression/mania) group, ICD-10/9 codes (88):**

| F30.10 | Manic behavior |
| --- | --- |
| F30.11 | Manic d/o, single episode, mild |
| F30.12 | Manic d/o, single episode, moderate |
| F30.13 | Manic d/o, single episode, severe |
| F30.2 | Severe bipolar I d/o, single manic episode with psychotic features |
| F30.8 | Manic d/o, atypical |
| F30.9 | Manic psychosis |
| F31.0 | Bipolar d/o, most recent episode hypomanic |
| F31.10 | Manic bipolar I d/o |
| F31.11 | Mild Manic bipolar I d/o |
| F31.12 | Moderate Manic bipolar I d/o |
| F31.13 | Severe Manic bipolar I d/o w/o psychotic features |
| F31.2 | Severe Manic bipolar I d/o with psychotic features |
| F31.30 | Bipolar I d/o, most recent episode depressed |
| F31.31 | Mild depressed bipolar I d/o |
| F31.32 | Moderate depressed bipolar I d/o |
| F31.4 | Severe depressed bipolar I d/o w/o psychotic features |
| F31.5 | Severe depressed bipolar I d/o with psychotic features |
| F31.60 | Mixed bipolar I d/o |
| F31.61 | Mild mixed bipolar I d/o |
| F31.62 | Moderate mixed bipolar I d/o |
| F31.63 | Severe mixed bipolar I d/o w/o psychotic features |
| F31.64 | Severe mixed bipolar I d/o with psychotic features |
| F31.81 | Bipolar II d/o |
| F31.89 | Severe bipolar d/o with psychotic features |
| F31.9 | Manic-depressive psychosis, unspecified; bipolar depression |
| F32.0 | Major depressive disorder, single episode, mild |
| F32.1 | Major depressive disorder, single episode, moderate |
| F32.3 | Major depressive disorder, single episode, severe with psychotic features |
| F32.2 | Major depressive disorder, single episode, severe without psychotic features |
| F32.9, R45.851 | Depression with suicidal ideation |
| F32.9 | Major depressive disorder, single episode, unspecified |
| F33.0 | Major depressive disorder, recurrent, mild |
| F33.1 | Major depressive disorder, recurrent, moderate |
| F33.2 | Major depressive disorder, recurrent severe without psychotic features |
| F33.3 | Major depressive disorder, recurrent, severe with psychotic symptoms |
| F33.9 | Major depressive disorder, recurrent, unspecified |
| F34.0 | Cyclothymic disorder |
| F34.1 | Dysthymic disorder |
| F34.8 | Other persistent mood [affective] disorders |
| F34.81 | Disruptive mood dysregulation disorder |
| F34.89 | Other specified persistent mood disorders |
| F34.9 | Persistent mood [affective] disorder, unspecified |
| F39 | Affective psychosis/mood disorder |
|  |  |
| 296.00 | Bipolar I disorder, single manic episode, unspecified |
| 296.01 | Bipolar I disorder, single manic episode, mild |
| 296.02 | Bipolar I disorder, single manic episode, moderate |
| 296.03 | Bipolar I disorder, single manic episode, severe, without mention of psychotic behavior |
| 296.04 | Bipolar I disorder, single manic episode, severe, specified as with psychotic behavior |
| 296.10 | Manic affective disorder, recurrent episode, unspecified |
| 296.11 | Manic affective disorder, recurrent episode, mild |
| 296.12 | Manic affective disorder, recurrent episode, moderate |
| 296.13 | Manic affective disorder, recurrent episode, severe, without mention of psychotic behavior |
| 296.14 | Manic affective disorder, recurrent episode, severe, specified as with psychotic behavior |
| 296.20 | Major depressive affective disorder, single episode, unspecified |
| 296.21 | Major depressive affective disorder, single episode, mild |
| 296.22 | Major depressive affective disorder, single episode, moderate |
| 296.23 | Major depressive affective disorder, single episode, severe, without mention of psychotic behavior |
| 296.24 | Major depressive affective disorder, single episode, severe, specified as with psychotic behavior |
| 296.30 | Major depressive affective disorder, recurrent episode, unspecified |
| 296.31 | Major depressive affective disorder, recurrent episode, mild |
| 296.32 | Major depressive affective disorder, recurrent episode, moderate |
| 296.33 | Major depressive affective disorder, recurrent episode, severe, without mention of psychotic behavior |
| 296.34 | Major depressive affective disorder, recurrent episode, severe, specified as with psychotic behavior |
| 296.40 | Bipolar I disorder, most recent episode (or current) manic, unspecified |
| 296.41 | Bipolar I disorder, most recent episode (or current) manic, mild |
| 296.42 | Bipolar I disorder, most recent episode (or current) manic, moderate |
| 296.43 | Bipolar I disorder, most recent episode (or current) manic, severe, without mention of psychotic behavior |
| 296.44 | Bipolar I disorder, most recent episode (or current) manic, severe, specified as with psychotic behavior |
| 296.50 | Bipolar I disorder, most recent episode (or current) depressed, unspecified |
| 296.51 | Bipolar I disorder, most recent episode (or current) depressed, mild |
| 296.52 | Bipolar I disorder, most recent episode (or current) depressed, moderate |
| 296.53 | Bipolar I disorder, most recent episode (or current) depressed, severe, without mention of psychotic behavior |
| 296.54 | Bipolar I disorder, most recent episode (or current) depressed, severe, specified as with psychotic behavior |
| 296.60 | Bipolar I disorder, most recent episode (or current) mixed, unspecified |
| 296.61 | Bipolar I disorder, most recent episode (or current) mixed, mild |
| 296.62 | Bipolar I disorder, most recent episode (or current) mixed, moderate |
| 296.63 | Bipolar I disorder, most recent episode (or current) mixed, severe, without mention of psychotic behavior |
| 296.64 | Bipolar I disorder, most recent episode (or current) mixed, severe, specified as with psychotic behavior |
| 296.7 | Bipolar I disorder, most recent episode (or current) unspecified |
| 296.80 | Bipolar disorder, unspecified |
| 296.81 | Atypical manic disorder |
| 296.82 | Atypical depressive disorder |
| 296.89 | Other bipolar disorders |
| 296.9 | Other and unspecified episodic mood disorder |
| 296.90 | Unspecified episodic mood disorder |
| 296.99 | Other specified episodic mood disorder |
|  |  |
| 311 | Depressive disorder, not elsewhere classified |

**Psychotic disorder group, ICD-10/9 codes (66):**

| F20.0 | Paranoid schizophrenia (+subchronic condition with acute exacerbation) |
| --- | --- |
| F20.1 | Disorganized schizophrenia (+ subchronic condition with acute exacerbation) |
| F20.2 | Catatonic schizophrenia (+ subchronic condition with acute exacerbation) |
| F20.3 | Undifferentiated schizophrenia (+ subchronic condition with acute exacerbation) |
| F20.5 | Residual schizophrenia (+ subchronic condition with acute exacerbation) |
| F20.89 | Acute schizophrenic episode (+ subchronic condition with acute exacerbation) |
| F20.9 | Schizophrenia, unspecified |
| F21 | Latent schizophrenia/schizotypal personality d/o |
| F22 | Paranoid psychosis |
| F23 | Brief psychotic disorder |
| F24 | Induced psychosis |
| F25.0 | Schizoaffective d/o, bipolar type |
| F25.1 | Schizoaffective d/o, depressed type |
| F25.8 | Other Schizoaffective d/o |
| F25.9 | Schizo-affective psychosis |
| F29 | Psychotic d/o |
|  |  |
| 295.00 | Simple type schizophrenia, unspecified |
| 295.01 | Simple type schizophrenia, subchronic |
| 295.02 | Simple type schizophrenia, chronic |
| 295.03 | Simple type schizophrenia, subchronic with acute exacerbation |
| 295.04 | Simple type schizophrenia, chronic with acute exacerbation |
| 295.10 | Disorganized type schizophrenia, unspecified |
| 295.11 | Disorganized type schizophrenia, subchronic |
| 295.12 | Disorganized type schizophrenia, chronic |
| 295.13 | Disorganized type schizophrenia, subchronic with acute exacerbation |
| 295.14 | Disorganized type schizophrenia, chronic with acute exacerbation |
| 295.20 | Catatonic type schizophrenia, unspecified |
| 295.21 | Catatonic type schizophrenia, subchronic |
| 295.22 | Catatonic type schizophrenia, chronic |
| 295.23 | Catatonic type schizophrenia, subchronic with acute exacerbation |
| 295.24 | Catatonic type schizophrenia, chronic with acute exacerbation |
| 295.30 | Paranoid type schizophrenia, unspecified |
| 295.31 | Paranoid type schizophrenia, subchronic |
| 295.32 | Paranoid type schizophrenia, chronic |
| 295.33 | Paranoid type schizophrenia, subchronic with acute exacerbation |
| 295.34 | Paranoid type schizophrenia, chronic with acute exacerbation |
| 295.40 | Schizophreniform disorder, unspecified |
| 295.41 | Schizophreniform disorder, subchronic |
| 295.42 | Schizophreniform disorder, chronic |
| 295.43 | Schizophreniform disorder, subchronic with acute exacerbation |
| 295.44 | Schizophreniform disorder, chronic with acute exacerbation |
| 295.50 | Latent schizophrenia, unspecified |
| 295.51 | Latent schizophrenia, subchronic |
| 295.52 | Latent schizophrenia, chronic |
| 295.53 | Latent schizophrenia, subchronic with acute exacerbation |
| 295.54 | Latent schizophrenia, chronic with acute exacerbation |
| 295.60 | Schizophrenic disorders, residual type, unspecified |
| 295.61 | Schizophrenic disorders, residual type, subchronic |
| 295.62 | Schizophrenic disorders, residual type, chronic |
| 295.63 | Schizophrenic disorders, residual type, subchronic with acute exacerbation |
| 295.64 | Schizophrenic disorders, residual type, chronic with acute exacerbation |
| 295.70 | Schizoaffective disorder, unspecified |
| 295.71 | Schizoaffective disorder, subchronic |
| 295.72 | Schizoaffective disorder, chronic |
| 295.73 | Schizoaffective disorder, subchronic with acute exacerbation |
| 295.74 | Schizoaffective disorder, chronic with acute exacerbation |
| 295.80 | Other specified types of schizophrenia, unspecified |
| 295.81 | Other specified types of schizophrenia, subchronic |
| 295.82 | Other specified types of schizophrenia, chronic |
| 295.83 | Other specified types of schizophrenia, subchronic with acute exacerbation |
| 295.84 | Other specified types of schizophrenia, chronic with acute exacerbation |
| 295.90 | Unspecified schizophrenia, unspecified |
| 295.91 | Unspecified schizophrenia, subchronic |
| 295.92 | Unspecified schizophrenia, chronic |
| 295.93 | Unspecified schizophrenia, subchronic with acute exacerbation |
| 295.94 | Unspecified schizophrenia, chronic with acute exacerbation |

**Substance use related group, ICD-10/9 codes (159):**

| F10.10 | ETOH abuse |
| --- | --- |
| F10.120 | Very severe alcohol intoxication, uncomplicated /ETOH abuse with intoxication |
| F10.130 | Alcohol abuse with withdrawal w/o complication |
| F10.131 | Alcohol abuse with withdrawal delirium |
| F10.139 | Alcohol abuse with withdrawal, unspecified |
| F10.180 | Alcohol abuse with alcohol-induced anxiety disorder |
| F10.14 | Alcohol abuse with alcohol-induced mood disorder |
| F10.150 | Alcohol abuse with alcohol-induced psychotic disorder with delusions |
| F10.151 | Alcohol abuse with alcohol-induced psychotic disorder with hallucinations |
| F10.159 | Alcohol abuse with alcohol-induced psychotic disorder, unspecified |
| F10.121 | Alcohol abuse with intoxication delirium |
| F10.129 | Alcohol abuse with intoxication, unspecified |
| F10.188 | Alcohol abuse with other alcohol-induced disorder |
| F10.19 | Alcohol abuse with unspecified alcohol-induced disorder |
| F10.20 | Alcoholism/Alcohol abuse |
| F10.280 | Alcohol dependence with alcohol-induced anxiety disorder |
| F10.24 | Alcohol dependence with alcohol-induced mood disorder |
| F10.250 | Alcohol dependence with alcohol-induced psychotic disorder with delusions |
| F10.251 | Alcohol dependence with alcohol-induced psychotic disorder with hallucinations |
| F10.259 | Alcohol dependence with alcohol-induced psychotic disorder, unspecified |
| F10.220 | Alcohol dependence with intoxication, uncomplicated |
| F10.221 | Alcohol dependence with intoxication delirium |
| F10.229 | Alcohol dependence with intoxication, unspecified |
| F10.230 | Alcohol withdrawal, uncomplicated |
| F10.231 | Alcohol dependence with withdrawal delirium (DTs) |
| F10.239 | Alcohol dependence with withdrawal, unspecified |
| F10.980 | Alcohol use, unspecified with alcohol-induced anxiety disorder |
| F10.94 | Alcohol use, unspecified with alcohol-induced mood disorder |
| F10.950 | Alcohol use, unspecified with alcohol-induced psychotic disorder with delusions |
| F10.951 | Alcohol use, unspecified with alcohol-induced psychotic disorder with  hallucinations |
| F10.959 | Alcohol use, unspecified with alcohol-induced psychotic disorder, unspecified |
| F10.920 | Alcohol intoxication, uncomplicated |
| F10.921 | Alcohol use, unspecified with intoxication delirium |
| F10.929 | Alcohol use, unspecified with intoxication, unspecified |
| F10.96 | Wernicke-Korsakoff syndrome (alcoholic) |
| F10.99 | Alcohol use, unspecified with unspecified alcohol-induced disorder |
|  |  |
| F11.10 | Opioid abuse |
| F11.120 | Uncomplicated opioid abuse with intoxication |
| F11.121 | Opioid abuse with intoxication delirium |
| F11.129 | Opioid abuse with intoxication |
| F11.13 | Opioid abuse with withdrawal |
| F11.14 | Opioid abuse with opioid-induced mood disorder |
| F11.150 | Opioid abuse with opioid-induced psychotic disorder with delusions |
| F11.151 | Opioid abuse with opioid-induced psychotic disorder with hallucinations |
| F11.159 | Opioid abuse with opioid-induced psychotic disorder, unspecified |
| F11.188 | Opioid abuse with other opioid-induced disorder |
| F11.19 | Opioid abuse with unspecified opioid-induced disorder |
| F11.221 | Opioid dependence with intoxication delirium |
| F11.24 | Opioid dependence with opioid-induced mood disorder |
| F11.250 | Opioid dependence with opioid-induced psychotic disorder with delusions |
| F11.251 | Opioid dependence with opioid-induced psychotic disorder with hallucinations |
| F11.259 | Opioid dependence with opioid-induced psychotic disorder, unspecified |
| F11.288 | Opioid dependence with other opioid-induced disorder |
| F11.29 | Opioid dependence with unspecified opioid-induced disorder |
| F11.921 | Opioid use, unspecified with intoxication delirium |
| F11.959 | Opioid use, unspecified with opioid-induced psychotic disorder, unspecified |
|  |  |
| F12.180 | Cannabis abuse with cannabis-induced anxiety disorder |
| F12.121 | Cannabis abuse with intoxication delirium |
| F12.150 | Cannabis abuse with psychotic disorder with delusions |
| F12.151 | Cannabis abuse with psychotic disorder with hallucinations |
| F12.159 | Cannabis abuse with psychotic disorder, unspecified |
|  |  |
| F13.121 | Sedative, hypnotic or anxiolytic abuse with intoxication delirium |
| F13.188 | Sedative, hypnotic or anxiolytic abuse with other sedative, hypnotic or anxiolytic induced disorder |
| F13.180 | Sedative, hypnotic or anxiolytic abuse with sedative, hypnotic or anxiolytic induced anxiety disorder |
| F13.14 | Sedative, hypnotic or anxiolytic abuse with sedative, hypnotic or anxiolytic induced mood disorder |
| F13.151 | Sedative, hypnotic or anxiolytic abuse with sedative, hypnotic or anxiolytic induced psychotic disorder |
| F13.221 | Sedative, hypnotic or anxiolytic dependence with intoxication delirium |
| F13.239 | Sedative, hypnotic or anxiolytic dependence with withdrawal, unspecified |
| F13.921 | Sedative, hypnotic or anxiolytic use, unspecified with intoxication delirium |
| F14.180 | Cocaine abuse with cocaine-induced anxiety disorder |
| F14.14 | Cocaine abuse with cocaine-induced mood disorder |
| F14.150 | Cocaine abuse with cocaine-induced psychotic disorder with delusions |
| F14.151 | Cocaine abuse with cocaine-induced psychotic disorder with hallucinations |
| F14.159 | Cocaine abuse with cocaine-induced psychotic disorder, unspecified |
| F14.121 | Cocaine abuse with intoxication with delirium |
| F14.19 | Cocaine abuse with unspecified cocaine-induced disorder |
|  |  |
| F15.10 | Amphetamine/stimulant abuse |
|  |  |
| F16.10 | Hallucinogen/PCP abuse |
|  |  |
| F18.10 | Inhalant abuse |
| F18.180 | Inhalant abuse with inhalant-induced anxiety disorder |
| F18.14 | Inhalant abuse with inhalant-induced mood disorder |
| F18.150 | Inhalant abuse with inhalant-induced psychotic disorder with delusions |
| F18.151 | Inhalant abuse with inhalant-induced psychotic disorder with hallucinations |
| F18.159 | Inhalant abuse with inhalant-induced psychotic disorder, unspecified |
| F18.121 | Inhalant abuse with intoxication delirium |
|  |  |
| F19.10 | Polysubstance abuse |
| F19.20 | Combinations of drug dependence excluding opioid type drug, abuse |
| F19.94 | Other psychoactive substance use, unspecified with psychoactive substance-induced mood disorder |
|  |  |
| 291 | Alcohol-induced mental disorders |
| 291.3 | Alcohol-induced psychotic disorder with hallucinations |
| 291.5 | Alcohol-induced psychotic disorder with delusions |
| 291.81 | Alcohol withdrawal |
| 291.89 | Other alcohol-induced mental disorders |
| 291.9 | Unspecified alcohol-induced mental disorders |
| 303.00 | Acute alcoholic intoxication in alcoholism, unspecified |
| 303.01 | Acute alcoholic intoxication in alcoholism, continuous |
| 303.02 | Acute alcoholic intoxication in alcoholism, episodic |
| 303.90 | Other and unspecified alcohol dependence, unspecified |
| 303.91 | Other and unspecified alcohol dependence, continuous |
| 303.92 | Other and unspecified alcohol dependence, episodic |
| 304.20 | Cocaine dependence, unspecified |
| 304.21 | Cocaine dependence, continuous |
| 304.22 | Cocaine dependence, episodic |
| 304.30 | Cannabis dependence, unspecified |
| 304.31 | Cannabis dependence, continuous |
| 304.32 | Cannabis dependence, episodic |
| 304.40 | Amphetamine and other psychostimulant dependence, unspecified |
| 304.41 | Amphetamine and other psychostimulant dependence, continuous |
| 304.42 | Amphetamine and other psychostimulant dependence, episodic |
| 304.50 | Hallucinogen dependence, unspecified |
| 304.51 | Hallucinogen dependence, continuous |
| 304.52 | Hallucinogen dependence, episodic |
| 304.60 | Other specified drug dependence, unspecified |
| 304.61 | Other specified drug dependence, continuous |
| 304.62 | Other specified drug dependence, episodic |
| 304.70 | Combinations of opioid type drug with any other drug dependence, unspecified |
| 304.71 | Combinations of opioid type drug with any other drug dependence, continuous |
| 304.72 | Combinations of opioid type drug with any other drug dependence, episodic |
| 304.80 | Combinations of drug dependence excluding opioid type drug, unspecified |
| 304.81 | Combinations of drug dependence excluding opioid type drug, continuous |
| 304.82 | Combinations of drug dependence excluding opioid type drug, episodic |
| 304.90 | Unspecified drug dependence, unspecified |
| 304.91 | Unspecified drug dependence, continuous |
| 304.92 | Unspecified drug dependence, episodic |
| 305.00 | Alcohol abuse, unspecified |
| 305.01 | Alcohol abuse, continuous |
| 305.02 | Alcohol abuse, episodic |
| 305.20 | Cannabis abuse, unspecified |
| 305.21 | Cannabis abuse, continuous |
| 305.22 | Cannabis abuse, episodic |
| 305.30 | Hallucinogen abuse, unspecified |
| 305.31 | Hallucinogen abuse, continuous |
| 305.32 | Hallucinogen abuse, episodic |
| 305.40 | Sedative, hypnotic or anxiolytic abuse, unspecified |
| 305.41 | Sedative, hypnotic or anxiolytic abuse, continuous |
| 305.42 | Sedative, hypnotic or anxiolytic abuse, episodic |
| 305.50 | Opioid abuse, unspecified |
| 305.51 | Opioid abuse, continuous |
| 305.52 | Opioid abuse, episodic |
| 305.60 | Cocaine abuse, unspecified |
| 305.61 | Cocaine abuse, continuous |
| 305.62 | Cocaine abuse, episodic |
| 305.70 | Amphetamine or related acting sympathomimetic abuse, unspecified |
| 305.71 | Amphetamine or related acting sympathomimetic abuse, continuous |
| 305.72 | Amphetamine or related acting sympathomimetic abuse, episodic |
| 305.80 | Antidepressant type abuse, unspecified |
| 305.81 | Antidepressant type abuse, continuous |
| 305.82 | Antidepressant type abuse, episodic |
| 305.90 | Other, mixed, or unspecified drug abuse, unspecified |
| 305.91 | Other, mixed, or unspecified drug abuse, continuous |
| 305.92 | Other, mixed, or unspecified drug abuse, episodic |
|  |  |
| K70.0 | Alcoholic fatty liver |
| K70.11 | Acute alcoholic liver disease, Alcoholic hepatitis with ascites |
| K70.40 | Alcoholic liver failure |
| K70.41 | Alcoholic hepatic failure with coma |
| K70.9 | Alcohol liver damage |
|  |  |
| 571.0 | Alcoholic fatty liver |
| 571.1 | Acute alcoholic hepatitis |
| 571.3 | Alcohol liver damage, unspecified |

**Other group, ICD-10/9 codes (208):**

| F50.0 | Anorexia nervosa, unspecified |
| --- | --- |
| F50.01 | Anorexia nervosa, restricting type |
| F50.02 | Anorexia nervosa with bulimia |
| F50.2 | Bulimia nervosa |
| F50.81 | Extreme binge-eating d/o |
| F50.89 | Cyclical vomiting, psychogenic |
| F50.9 | Eating d/o |
| F53.0 | Severe depressive episode with and w/o psychotic symptoms in postpartum |
| F53.1 | Puerperal psychosis |
| F60.0 | Paranoid personality d/o |
| F60.1 | Schizoid personality |
| F60.2 | Psychopathic personality d/o |
| F60.3 | Borderline personality d/o |
| F60.4 | Histrionic personality |
| F60.5 | OCD personality d/o |
| F60.6 | Anxious personality d/o |
| F60.7 | Dependent personality d/o |
| F60.81 | Narcissistic personality d/o |
| F 60.89 | Passive-aggressive personality |
| F60.9 | Personality, masochistic |
| F68.10 | Factitious d/o |
|  |  |
| 301.0 | Paranoid personality disorder |
| 301.10 | Affective personality disorder, unspecified |
| 301.11 | Chronic hypomanic personality disorder |
| 301.12 | Chronic depressive personality disorder |
| 301.13 | Cyclothymic disorder |
| 301.20 | Schizoid personality disorder, unspecified |
| 301.22 | Schizotypal personality disorder |
| 301.3 | Explosive personality disorder |
| 301.4 | Obsessive-compulsive personality disorder |
| 301.50 | Histrionic personality disorder, unspecified |
| 301.51 | Chronic factitious illness with physical symptoms |
| 301.59 | Other histrionic personality disorder |
| 301.6 | Dependent personality disorder |
| 301.7 | Antisocial personality disorder |
| 301.81 | Narcissistic personality disorder |
| 301.82 | Avoidant personality disorder |
| 301.83 | Borderline personality disorder |
| 301.84 | Passive-aggressive personality |
| 301.89 | Other personality disorders |
| 301.9 | Unspecified personality disorder |
|  |  |
| 307.1 | Anorexia nervosa |
| 307.50 | Eating d/o, unspecified |
| 307.51 | Bulimia nervosa |
| 307.59 | Other disorders of eating |
| 307.80 | Psychogenic pain, site unspecified |
|  |  |
| T40.0X1A  T40.0X2A  T40.0X4A  T40.1X4A  T40.1X1A  T40.1X2A  T40.2X1A  T40.2X2A  T40.2X4A  T40.3X2A  T40.3X4A  T40.411A  T40.412A  T40.414A  T40.422A  T40.424A  T40.492A  T40.494A  T40.5X1A  T40.601A  T40.602A  T40.604A  T40.692A  T40.694A  T40.7X1A  T40.7X2A  T40.7X4A  T40.8X1A  T40.8X2A  T40.8X4A  T40.901A  T40.902A  T40.904A  T40.991A  T40.992A  T40.994A  T43.601A  T43.602A  T43.604A  T43.612A  T43.614A  T43.621A  T43.622A  T43.624A  T43.632A  T43.634A  T43.641A  T43.642A  T43.644A  T43.691A  T43.692A  T43.694A | All opioid, stimulants poisoning (illicits – all intents (exc assault), initial encounter; opioids- intentional, undetermined intent, initial encounter) |
| T36.0X2A  T36.2X2A  T36.4X2A  T36.5X2A  T36.6X2A  T36.7X2A  T36.8X2A  T36.92XA  T37.0X2A  T37.1X2A  T37.2X2A  T37.3X2A  T37.4X2A  T37.5X2A  T37.8X2A  T37.92XA  T38.0X2A  T38.1X2A  T38.2X2A  T38.3X2A  T38.802A  T39.012A  T39.092A  T39.1X2A  T39.312A  T39.8X2A  T39.92XA  T41.0X2A  T42.0X2A  T42.1X2A  T42.3X2A T42.4X2A  T42.5X2A  T42.72XA  T42.82XA  T43.012A  T43.1X2A  T43.202A  T43.212A  T43.222A  T43.292A  T43.3X2A  T43.4X2A  T43.502A  T43.8X2A  T43.92XA  T44.0X2A  T44.5X2A  T44.6X2A  T44.7X2A  T44.8X2A  T44.902A  T45.0X2A  T46.0X2A  T46.2X2A  T46.4X2A  T46.5X2A  T46.902A  T47.0X2A  T47.12XA  T47.2X2A  T47.4X2A  T47.8X2A  T47.92XA  T50.0X2A  T50.1X2A  T50.2X2A  T50.4X2A  T50.901A  T50.911A  T50.912A  T50.914A | Pharmaceutical poisoning (intentional, initial encounter) |
| T51.0X2A | Poisoning by ingestion of illicit alcohol, intentional self harm |
| T51.1X2A | Methanol poisoning, intentional self harm |
| T51.2X2A | Isopropyl poisoning, intentional self harm |
| T51.8X2A | Toxic effect of other alcohols, intentional self harm |
| T51.92XA | Suicide attempt by alcohol poisoning |
| \| T54.3X2A \| \| --- \| | Poisoning, intentional self-harm by Alkali (caustic) |
| T14.91 | Suicide attempt |
| T14.91XA | Suicide attempt, initial encounter |
| T14.91XD | Suicide attempt, subsequent encounter |
| T50.902A | Suicidal overdose |
| T58.02XA | Suicide by poisoning by motor vehicle exhaust gas |
| T65.92XA | Suicidal deliberate poisoning by substance/chemical overdose |
| T71.162A | Suicide attempt by hanging, initial encounter |
| R45.851 | Suicidal ideation/thoughts |
| R45.89 | Suicidal behavior |
| X74.9XXA | Suicide by firearm |
|  |  |
| E950.0 | Suicide and self-inflicted poisoning by analgesics, antipyretics, and antirheumatics |
| E950.1 | Suicide and self-inflicted poisoning by barbiturates |
| E950.2 | Suicide and self-inflicted poisoning by other sedatives and hypnotics |
| E950.3 | Suicide and self-inflicted poisoning by tranquilizers and other psychotropic agents |
| E950.4 | Suicide and self-inflicted poisoning by other specified drugs and medicinal substances |
| E950.5 | Suicide and self-inflicted poisoning by unspecified drug or medicinal substance |
| E950.7 | Suicide and self-inflicted poisoning by corrosive and caustic |
| E950.9 | Suicide and self-inflicted poisoning by other and unspecified solid and liquid substances |
| E953.0 | Suicide and self-inflicted injury by hanging |
| E955.9 | Suicide and self-inflicted injury by firearms and explosives, unspecified |
| E955.0 | Suicide and self-inflicted injury by handgun |
| E958.9 | Suicide and self-inflicted injury by unspecified means |
|  |  |
| 965.00 | Poisoning by opium (alkaloids), unspecified |
| 965.01 | Poisoning by heroin |
| 969.6 | Poisoning by psychodysleptics (hallucinogens) |
| 969.70 | Poisoning by psychostimulant, unspecified |
| 969.72 | Poisoning by amphetamines |
| 969.79 | Poisoning by other psychostimulants |
| 969.8 | Poisoning by other specified psychotropic agents |
| 969.9 | Poisoning by unspecified psychotropic agent |
| 970.81 | Poisoning by cocaine |
| 970.89 | Poisoning by other central nervous system stimulants |
| 970.9 | Poisoning by unspecified central nervous system stimulant |
